# Tackling Carbapenem Resistance and the Imperative for One Health Strategies—Insights from the Portuguese Perspective

**DOI:** 10.3390/antibiotics13060557

**Published:** 2024-06-14

**Authors:** Inês Mó, Gabriela Jorge da Silva

**Affiliations:** 1 Faculty of Pharmacy, University of Coimbra, 3000-548 Coimbra, Portugal; inesfariamo@gmail.com; 2 CNC, Center for Neuroscience and Cell Biology, 3004-504 Coimbra, Portugal

**Keywords:** carbapenemases, One Health, epidemiology, antibiotic resistance, β-lactams

## Abstract

Carbapenemases, a class of enzymes specialized in the hydrolysis of carbapenems, represent a significant threat to global public health. These enzymes are classified into different Ambler’s classes based on their active sites, categorized into classes A, D, and B. Among the most prevalent types are IMI/NMC-A, KPC, VIM, IMP, and OXA-48, commonly associated with pathogenic species such as *Acinetobacter baumannii*, *Klebsiella pneumoniae*, and *Pseudomonas aeruginosa*. The emergence and dissemination of carbapenemase-producing bacteria have raised substantial concerns due to their ability to infect humans and animals (both companion and food-producing) and their presence in environmental reservoirs. Adopting a holistic One Health approach, concerted efforts have been directed toward devising comprehensive strategies to mitigate the impact of antimicrobial resistance dissemination. This entails collaborative interventions, highlighting proactive measures by global organizations like the World Health Organization, the Center for Disease Control and Prevention, and the Food and Agriculture Organization. By synthesizing the evolving landscape of carbapenemase epidemiology in Portugal and tracing the trajectory from initial isolated cases to contemporary reports, this review highlights key factors driving antibiotic resistance, such as antimicrobial use and healthcare practices, and underscores the imperative for sustained vigilance, interdisciplinary collaboration, and innovative interventions to curb the escalating threat posed by antibiotic-resistant pathogens. Finally, it discusses potential alternatives and innovations aimed at tackling carbapenemase-mediated antibiotic resistance, including new therapies, enhanced surveillance, and public awareness campaigns.

## 1. Introduction

The “Golden Age” of antibiotic development began with the discovery of penicillin in 1928 by Sir Alexander Fleming and reached its peak in the 1950s. Nowadays, antibiotics play one of the most important roles in the battle against infectious diseases [1]. As a result of this discovery, development, and overuse, the number of bacterial resistances started to increase, leading to the current antimicrobial resistance crisis [2].

Carbapenems belong to the β-lactam family and act by preventing cell wall formation. These have increased activity against Gram-negative bacteria when compared to others in the same group, being used in cases of infections caused by bacteria resistant to other β-lactams, and other antibiotics like fluoroquinolones and aminoglycosides [3,4]. They should be used as the last treatment option in cases of resistance, or multidrug resistance, infections that do not respond to other antibiotics, such as those caused by *Pseudomonas aeruginosa*, *Acinetobacter baumannii,* and *Enterobacteriales* [5]. They can also be applied to treat both aerobic and anaerobic infections; strains of *pneumococci* not susceptible to penicillin [6,7].

The use of antibiotics to treat infections can lead to the development and spread of resistance, in which, through selection pressure, mutant strains can prevail and spread [8,9,10]. Concerning carbapenems, the main described resistance mechanism is the production of specialized enzymes called carbapenemases that can inhibit the action of the antibiotic by hydrolyzing their β-lactam ring [11,12].

The spread of carbapenemase-producing bacteria has been increasing considerably through the years [13]. Among the European countries, Portugal is a country where the carbapenem resistance remains high and with specific concerns, such as the increase of the carbapenem-resistant *Klebsiella pneumoniae* (10.29% in 2022) [14,15].

The strategy to combat this problem is complex and quite demanding, since the inappropriate or unnecessary use of antibiotics occurs in various sectors (human, animal, and agriculture). Therefore, in conjunction with several entities, the “One Health” initiative was created and implemented in numerous countries, including Portugal. In this approach, human and animal health, as well as the agriculture, animal-food production, and environment sectors of the various countries, work together in an endeavor to address the challenges arising from antimicrobial resistance [16,17].

In this review, we explore the evolution of carbapenem-resistant bacteria, particularly emphasizing the main mechanism of resistance, carbapenemase classification, and their prevalence across diverse sectors, emphasizing a One Health perspective, by using Portugal as a model to illustrate these concepts in practice.

## 2. The Carbapenems: Mechanisms of Action and Resistance

Carbapenems are β-lactam antibiotics. The first carbapenem approved as an antibacterial drug was imipenem in 1985, and it is currently used in combination with cilastatin, a dehydropeptidase-1 (DHP-1) inhibitor, to avoid the inactivation of imipenem and metabolite-associated nephrotoxicity [18]. Subsequently, other carbapenems resistant to this renal enzyme were developed, such as meropenem and ertapenem in 1996 and 2001, respectively [19].

**Figure 1:**
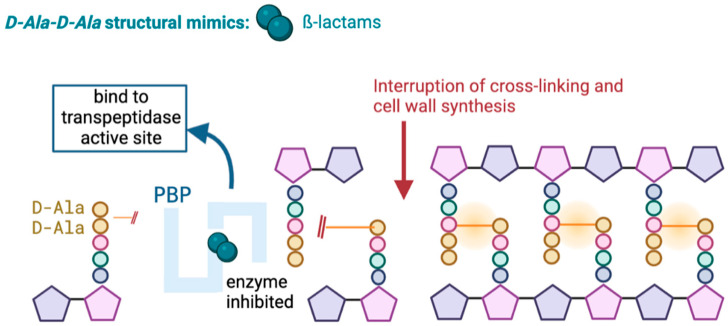
Mechanism of action of β-lactams.

Carbapenems, like other β-lactam antibiotics, act by inhibiting the synthesis of the peptidoglycan of the bacterial cell wall. This occurs through irreversible binding of the β-lactam to penicillin-binding proteins (PBP) at the active site of the transpeptidase, where typically the terminal amino acid residues would bind, leading to the death of the cell wall (Figure 1) [19,20].

**Figure 2:**
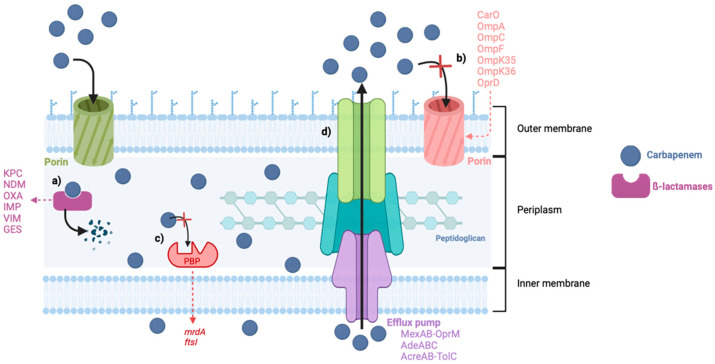
Mechanisms of carbapenem resistance in Gram-negative bacteria. (a) Production of carbapenemases. (b) Decreased permeability of the porins. (c) Reduced affinity of the PBPs. (d) Increased activity of the efflux pumps. Abbreviations: KPC: Klebsiella pneumoniae carbapenemases, NDM: New Delhi Metallo-β-lactamases, OXA: Oxacillin-hydrolyzing carbapenemases, IMP: Imipenem hydrolyzing β-lactamase, VIM: Verona Integron-encoded Metallo-β-lactamases, GES: Guiana extended spectrum.

Carbapenem resistance can emerge by different mechanisms, like decreased outer membrane permeability due to mutations on the outer membrane proteins (OMPs), decreased affinity of the PBPs, and efflux pump overexpression, but the most frequently reported is the production of carbapenemases (Figure 2) [21]. The most common and threatening bacteria resistant to carbapenems are *Acinetobacter baumannii, Pseudomonas aeruginosa,* and some members of the *Enterobacteriaceae* family, such as *Klebsiella pneumoniae* [22]. These are included in the Ia priority list of antibiotic-resistant bacteria in need of new antibiotics to alert for research and development, published by the World Health Organization (WHO) in 2017, where carbapenem-resistant bacteria were placed at the top of this list, considered critical [23].

The production of carbapenemases represents the main mechanism of carbapenem resistance and, alongside carbapenems, is capable of hydrolyzing other β-lactam antibiotics such as penicillin, cephalosporins, and aztreonam [24].

## 3. Carbapanemases

To date, there are more than 8000 β-lactamases described, and their classification is based on their functional features or on the amino acid sequences [25,26,27]. In 1980, Ambler established a structural classification for these enzymes, initially into two classes, A and B, based on the information obtained from amino acid sequencing and also according to the type of hydrolytic mechanism. Later, two more classes, C and D, were added to this classification system in order to better distinguish the β-lactamases that previously belonged to class A [25,28]. Regarding functional characteristics, Bush, Jacoby, and Medeiros proposed a new classification system in 1995, differentiating the β-lactamases into three major groups according to their substrate and inhibitor profiles, with additional subgroups added in 2010 by Bush and Jacoby [27,29]. Although class C β-lactamases were the first to be identified, they are not usually considered to possess carbapenemase activity [30]. However, this class of β-lactamases can be associated with carbapenem resistance when their overexpression is combined with other resistance mechanisms that lead to reduced permeability, such as porin loss or mutations [28].

Carbapenemases can belong to structural classes A, B, or D of the Ambler classification, or to the functional groups 2df, 2f, or 3 [31].

Carbapenemases that belong to classes A and D are considered serine-β-lactamase because they have a serine residue at their active center, hydrolyzing the antibiotics by forming a covalent intermediate through the active serine group [19,32]. In Ambler class B, metallo-β-carbapenemases can hydrolyze β-lactams due to their catalytic activity, provided by the presence of one or more zinc ions in the active site [33]. They are inhibited by metal chelators such as ethylenediaminetetraacetic acid (EDTA). Their genetic location is diverse and has an impact on their dissemination. The genes that lead to the production of these enzymes can be located on chromosomes or plasmids [34]. Plasmid-borne carbapenemases are much easier to spread [35]. Table 1 shows the Ambler classification of carbapenemases and the corresponding Bush group, as well as their genetic location and first identification.

**Table 1:** Carbapenemase Classification and Characterization.

Ambler Class	Bush’s Classification	Gene Location	Carbapenemase	First Identification
**A**	2f	Chromosome	SME (*Serratia marcescens* enzyme)	*Serratia marcescens* (UK, 1982) [36]
			NMC-A (Non-metalloenzyme carbapenemase-A)	*E. clocae* (France, 1990) [37]
			IMI (Imipenem-hydrolyzing β-lactamase)	*Enterobacter clocae* (USA, 1984) [38]
			SFC (*Serratia fonticola* carbapenemase)	*Serratia fonticola* (Portugal, 2004) [39]
	2e	Plasmid	KPC (*Klebsiella pneumoniae* carbapenemase)	*Klebsiella pneumonia* (USA, 1996) [40]
			GES (Guiana extended spectrum)	*P. aeruginosa* (South Africa, 2000) [41]
**B**	3	Plasmid	IMP (Imipenemase)	*Serratia marcescens* (Japan, 1991) [42]
			VIM (Verona integron-encoded metallo-β-lactamase)	*P. aeruginosa* (Italy, 1997) [43]
			SPM (Sao Paulo imipenemase)	*P. aeruginosa* (Brazil, 1997) [44]
			GIM (German imipenemase)	*P. aeruginosa* (Germany, 2002) [45]
			NDM (New Delhi metallo-β-lactamase)	*Klebsiella pneumoniae* (India, 2008) [46]
**D**	2df	Plasmid	OXA (Oxacillin-hydrolyzing carbapenemases)	OXA-48—*Klebsiella pneumonia* (Turkey, 2001) [47]

### 3.1. Class A Carbapenemases

This class of serine carbapenemases can hydrolyze a broad spectrum of β-lactams, including carbapenems, penicillins, cephalosporins, and aztreonam, but is inhibited by clavulanic acid and tazobactam [48,49,50].

The imipenem-hydrolyzing β-lactamase (IMI-1), not-metalloenzyme carbapenemase-A (NMC-A), *Serratia marcescens* enzyme (SME), and *Serratia fonticola* carbapenemase (SFC) are chromosomally encoded. The plasmid-encoded enzymes are *Klebsiella pneumoniae* carbapenemases (KPC) and Guiana extended spectrum (GES) [51].

Among all class A enzymes, it is relevant to highlight the KPCs, initially described in *Klebsiella pneumoniae* in 1996 and being distributed worldwide, with hydrolytic activity on all β-lactams [28,52]. KPCs have the potential to spread among human pathogens primarily through bacteria of the *Enterobacteriaceae* family [53].

### 3.2. Class B Carbapenemases

Class B carbapenemases, classified as metallo-β-lactamases (MBLs), are characterized by their ability to hydrolyze most β-lactam antibiotics, except aztreonam [31]. They can be inhibited by chelating agents such as EDTA and dipicolinic acid (DPA), but their activity can be reconstituted with the subsequent addition of zinc [54].

According to the need for one or two zinc molecules to have catalytic activity, MBLs have been divided into two subclasses: B1 and B3 need two zinc ions, while the B2 subclass only needs one ion in its active site [19].

Considering the three subclasses, B1 is currently of greater clinical relevance, partially because it is associated with mobile plasmids that facilitate the transfer of resistance genes between bacteria, primarily through *Pseudomonas* spp., *Acinetobacter* spp., and *Enterobacteriaceae* [6,55]. The most prevalent are Imipenemase (IMP), Verona integron-encoded metallo-β-lactamase (VIM,) and New Delhi metallo-β-lactamase (NDM) [56]. There have been reported cases of other MBLs, such as German Imipenemase (GIM) and Sao Paulo Metallo-β-lactamase (SPM) [44,57,58]. These families of enzymes have their genes encoded in a variety of integron structures, usually located in conjugative plasmids, which facilitates their dissemination [59]. IMPs and VIMs are mainly associated with *P. aeruginosa* and NDMs with *K. pneumoniae* [60,61]. Although NDMs have been discovered more recently, they are rapidly becoming a health problem due to their ability to spread rapidly around the world [62]. This concern also centers on the fact that no parameters concerning their prevalence have yet been established and the uncertainty of their incidence in the population other than health care institutions, which happens mostly in neonatal, pediatric, and surgical intensive care units [63].

### 3.3. Class D Carbapenemases

Class D carbapenemases were initially characterized as oxacillinases because they possessed hydrolytic activity for oxacillin, which gave them the classification of Oxacillin-hydrolyzing carbapenemases (OXAs). This class of enzymes is generally not inhibited by clavulanic acid, EDTA, tazobactam, or sulbactam, and many of them hydrolyze carbapenems with different efficiency [64]. The efficacy of their hydrolytic activity against carbapenems is diverse and sometimes difficult to defeat, but their production works in synergistic action with other resistance mechanisms [51].

These enzymes can be differentiated according to their spectrum of action into three groups, one of which is for OXAs with hydrolytic activity on carbapenems, namely the carbapenem-hydrolyzing class D β-lactamases (CHDLs) [65,66].

The most prevalent CHDL is OXA-48, with its first case reported in Turkey in 2001 on an isolate of *Klebsiella pneumoniae* [47]. OXA-48 is found mainly in *Klebsiella pneumoniae* and in numerous members of *Enterobacterales*, where it appears to be responsible for healthcare-associated infections. Concerning community-acquired infections, *Escherichia coli* appears to be the most common contributor [67].

## 4. Epidemiology of Carbapenemases in Portugal

In Portugal, the first carbapenemases were identified in the late nineties in the clinical setting, probable due to the increased use of carbapenems to fight infections associated with bacteria producers of extended-spectrum β-lactamases (ESBLs) and, at the same time, the emergence and endemicity of multidrug-resistant *A. baumannii* in Portuguese hospitals [68]. Since then, the rate of spread of carbapenem-resistant bacteria has been increasing, with sporadic cases and small outbreaks documented between 2010 and 2013 evolving into a significant increase in present days [69]. Table 2 shows all reported cases of carbapenemases in Portugal to date, according to their Ambler classification, the bacteria carrying the resistance enzymes, the source (clinical, animal, or environmental), and the year of the identification.

The first carbapenemases identified corresponded to MBLs but were mostly found in clinical settings with no relationship between them and did not have significant dissemination, especially IMP-5, which is endemic to this country [70,71,72,73]. They have also been found in environments under anthropogenic pressure, such as the identification of *Serratia fonticola* hydrolase 1 (SFH-1) in untreated drinking water and four isolates of NDM-1 in the Lis River in 2017 [39,74,75,76].

The other two classes of carbapenemases were identified later in Portugal, mainly in *Enterobacterales* and *Acinetobacter* spp., but since the first report, there has been greater concern regarding them [77,78,79].

Cases of class D carbapenemases have been sporadically reported, with OXA-48 being the most commonly found class D carbapenemase in clinical settings, with its first identification in 2013 in *E. coli* isolates in a Lisbon hospital [80]. There is a concern regarding OXAs due to their identification not only in hospitals but in animals as well, such as cats, dogs, and seagulls [81,82].

Regarding class A carbapenemases, the dissemination of carbapenemases is mainly found in *Klebsiella pneumoniae*, belonging to the KPC family. To date, around 200 different KPCs have been identified, with KPC-2 and KPC-3 being the two most reported carbapenemases in Portugal [26]. The first identification of KPC-3 was in 2009 in an isolate of *K. pneumoniae* at the Lisbon Hospital Center [83]. The first clinical isolate of KPC-2 in Portugal was identified in 2019 in *K. pneumoniae* isolates [13]. Previously, it was identified in *E. coli* in water samples collected from a river that crosses Santo Tirso in 2010, in the north of Portugal [84]. Although this class has been identified more recently in Portugal, the number of isolated cases has increased exponentially in recent years, all around the world. Its identification, although predominantly in a hospital environment, has also been found in rivers and animals, such as dogs and seagulls, which raises a greater concern for this specific public health problem [85].

**Table 2:** Carbapenemases identified in Portugal since their first identification until now.

**Class A**	**Bacteria**	**Source and Year**	**Reference**
**KPC type**	*E. coli*	River, 2021	[86]
	*K. pneumoniae*	Hospital, 2021–2022	[87]
		River, 2021	[86]
**KPC-2**	*E. coli*	River, 2010	[84]
		Hospital, 2014	[69]
	*K. pneumoniae*	Hospital, 2018–2019	[13]
		Seagulls, 2019	[85]
	*C. freundii*; *K. oxycota*	Seagulls, 2019	[85]
**KPC-3**	*E. aerogenes*; *E. cloacae*	Hospital, 2006–2013	[88]
	*K. pneumoniae*	Hospital, 2009; 2006–2013; 2013; 2013–2014; 2016; 2013–2018; 2017–2018; 2018; 2020; 2019–2021	[79,83,88,89,90,91,92,93,94,95]
		Community laboratories, 2014–2015	[96]
		Seagulls, 2019	[85]
		River, 2017	[74]
		Dogs, 2020	[97]
	*Klebsiella* spp.	Hospital, 2017–2018	[98]
	*P. aeruginosa*	Hospital, 2017–2018	[99]
	*E. coli*	Hospital, 2006–2013; 2017–2018	[88,93]
	*K. varicola*	Hospital, 2018	[94]
	*A. baumannii*	Hospital, 2018	[95]
	*K. oxycota*	WWTP, 2016–2019	[100]
	*Raoultella*; *Enterobacter*; *Citrobacter*	Urban pond, 2019	[101]
	*K. cryocrescens*	Hospital, 2019	[102]
**KPC-21**	*E. coli*	Hospital, 2014	[69]
**KPC-70**	*K. pneumoniae*	Hospital, 2019	[92]
**GES type**	*P. aeruginosa*	Hospital, 2017–2020	[103]
**GES-5**	*K. pneumoniae*	Hospital, 2009; 2012–2013; 2013; 2013–2018; 2019–2021	[69,88,91,92,104]
		Bivalve, 2022	[95]
	*Citrobacter*	River, 2017	[74]
	*C. freundii*	Seagulls, 2019	[85]
	*Raoultella*; *Enterobacter*; *Klebsiella* spp.	Urban pond, 2019	[101]
**GES-6**	*P. aeruginosa*	Hospital, 2012; 2015	[105,106]
	*E. cloacae*	Seagulls, 2019	[85]
**GES-13**	*P. aeruginosa*	Hospital, 2017–2018	[99]
**Class B MBLs**	**Bacteria**	**Source and Year**	**Reference**
**NDM-1**	*Providencia stuatii*	Hospital, 2015	[107]
	*Enterobacter*	River, 2017	[74]
	*K. pneumoniae*	Hospital, 2018–2019; 2019–2021; 2020	[79,92,108]
	*M. morganii*; *P. mirabilis*	Hospital, 2019	[109]
	*K. cryocrescens*	Hospital, 2019	[102]
	*E. coli*	Hospital, 2020	[79]
**NDM-5**	*E. coli*	Hospital, 2019	[110]
**IMP-5**	*A. baumanii*	Hospital, 1998	[68]
	*P. aeruginosa*	Hospital, 2001–2003	[72]
	*A. bereziniae*	Hospital, 2008–2012	[111]
**IMP-8**	*P. mendocina*	Hospital, 2005	[112]
	*K. pneumoniae*	Hospital, 2009	[104]
	*E. coli*	River, 2015	[71]
**IMP-22**	*K. pneumoniae*	Hospital, 2011–2012	[113]
**VIM-1**	*C. freundii*	Hospital, 2001–2002	[114]
	*E. coli*	River, 2015	[71]
	*Citrobacter*	Urban pond, 2019	[101]
**VIM-2**	*P. aeruginosa*	Hospital, 1995, 2003–2004	[70,115]
	*C. freundii*	Hospital, 2001–2002	[114]
	*K. oxycota*	Hospital, 2004	[116]
	*Morganella morgannii*	Hospital, 2004	[117]
	*K. pneumoniae*	Hospital, 2006–2013	[88]
	*Klebsiella* spp.	Hospital, 2017–2018	[98]
**VIM-34**	*K. pneumoniae*	Hospital, 2011–2012	[118]
	*E. coli*	River, 2015	[71]
**SFH-1**	*Serratia fonticola*	Untreated drinking water, 2003	[76]
**Class D**	**Bacteria**	**Source and Year**	**Reference**
**OXA-48**	*E. coli*	Hospital, 2013, 2017–2018	[80,93]
	*K. pneumoniae*	Hospital, 2017–2018; 2018; 2018–2019;	[13,94,98]
		Seagulls, 2019	[85]
**OXA-24/40**	*A. haemolyticus*; *A. baumannii*	Hospital, 2003–2004	[119]
**OXA-23**	*A. baumannii*	Hospital, 2006–2008	[120]
		Cat, 2014	[82]
**OXA-181**	*K. pneumoniae*	Hospital, 2016–2018; 2019–2021	[91,92]
		Cat, 2021	[121]
	*E. coli*	Seagulls, 2019	[85]
		Dog, 2020	[81]
**OXA-58**	*A. baumannii*	Hospital, 2005	[117]

Some isolates were also identified as co-producers of different carbapenemases, which potentially contributes to a higher rate of resistance. All the cases identified harbor *bla*_KPC_, with the carbapenemase on the rise in Portugal. The first isolates found are from a study in 2014, where *K. pneumoniae* co-harbor KPC-3 and GES-6 and were resistant to imipenem, meropenem, and ertapenem [69]. Later, the same bacteria were identified as co-producers of KPC-3 and GES-5, KPC-3 and OXA-48/181, and KPC-3 and VIM-2, with resistance to most of the β-lactams tested [91,92,98]. Other members of *Enterobacterales* were also isolated, such as *K. cryocrescens*, co-producing NDM-1 and KPC-3, resistant to all β-lactams, kanamycin, and trimethoprim-sulfamethoxazole [102]. In 2019, a case of *Citrobacter* co-harboring *bla*_VIM_ and *bla*_KPC_ was described for the first time in Portugal in an urban pond in Aveiro. Until the date of the study, it had only been reported in a clinical isolate in Spain. In the same study, other bacterial isolates of *Raoultella* and *Enterobacter* with the co-presence of KPC and GES were identified [101].

The knowledge of the epidemiology of carbapenemases in Portugal is a clear example that illustrates the intricate interface among various environments and how carbapenemases disseminate across them. The concomitant presence of these enzymes in humans as well as in the environment and in animals highlights the importance of tackling action in a unifying manner [122,123]. In this sense, several world organizations such as WHO, the World Organization for Animal Health (OIE), and the Food and Agriculture Organization (FAO) of the United Nations, worked on an initiative called One Health [124]. They aim to achieve the best health outcomes for everyone, from humans and animals to plants, always taking into consideration the interactions between all niches [2].

## 5. One Health in Portugal

Several countries have been adopting the One Health approach, including Portugal. This collaboration allows for greater, faster, and more reliable data collection and treatment, enabling better adaptation and implementation of the measures that have been developed [125].

In 2013, the Portuguese health organization, Direção Geral de Saúde (DGS), together with the National Health Institute Doctor Ricardo Jorge (INSA), launched guidelines to optimize the epidemiological surveillance system for antibiotic resistance, making it mandatory to report the identification of microorganisms of major concern, such as resistant *Staphylococcus aureus*, *Pseudomonas aeruginosa*, *Acinetobacter* spp., and *Enterobacterales* [126]. More recently, a new plan to deal with antimicrobial resistance started in 2019, which lasted until the end of 2023, with global objectives and proposed strategies as well as goals to be achieved by the end of this plan. Six main goals were established, which include improving knowledge about resistance, optimizing the use of antibiotics, improving epidemiological surveillance, environmental monitoring, and research [124]. Another major goal involves the commitment associated with economic measures and sustainable investments in diagnostic techniques, treatment of infections, and the development of new drugs [2].

Carbapenems belong to category A of the Antimicrobial Advice ad hoc Expert Group (AMEG) report on antibiotics for use in animals, meaning that they cannot be used in veterinary medicine. This expert group was established by the European Medicines Agency (EMA) to guide the impact of antibiotic use on animals. Exceptionally, they can be given to non-food-producing animals under strict legislation for this type of antibiotic [23].

On the environmental and animal sectors, better nutritional programs are intended, such as conditions within the animal yards with reduced livestock density, as well as establishing crop rotations, which are measures to be applied in order to reduce resistance to antibiotics [127].

Nevertheless, all the measures implemented need to be monitored in order to understand their efficiency and effectiveness in lowering antibiotic resistance.

### 5.1. Carbapenem Resistance in Portugal

**Figure 3:**
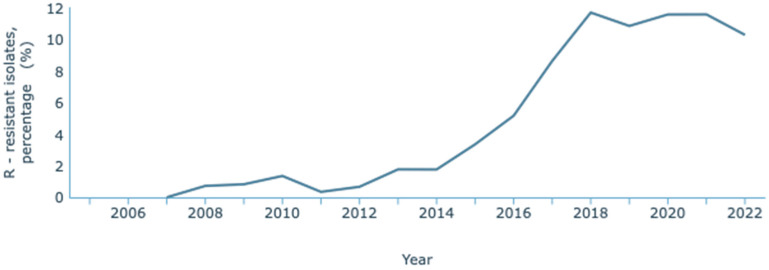
Rate of resistance to carbapenems by *K. pneumoniae*. Dataset provided by ECDC based on data provided by WHO and Ministries of Health from the affected countries (https://atlas.ecdc.europa.eu/public/index.aspx, accessed on 7 March 2024).

The most recent data concerning antimicrobial resistance in Portugal published by the WHO and the European Centre for Disease Prevention and Control (ECDC) is the 2021 report, issued in the year 2023 [128]. The ECDC, through the European Antimicrobial Resistance Surveillance Network (EARS-Net) and their database for the numbers of infectious diseases, the Surveillance Atlas of Infectious Diseases, has published resistance data until 2022 [129]. On this Atlas, it has been observed that *K. pneumoniae* resistance to carbapenems has increased over the years (Figure 3). Between 2007 and 2022, an increase from 0.8% to 10.9% of resistant infections was reported. These statistics refer to resistance to one or two carbapenems: meropenem and imipenem. INSA reported the data provided by the national laboratory that considered a third carbapenem, ertapenem. Its inclusion is relevant since it is a more sensitive marker of carbapenem resistance due to its lower stability to β-lactamases. Overall, between 2015 and 2022, there was an increase from 4.2% to 13.0% in carbapenem resistance [130].

**Figure 4:**
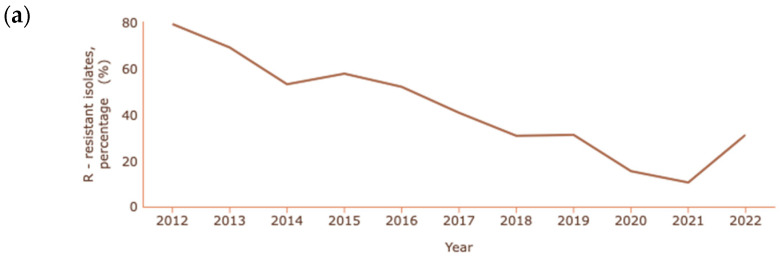

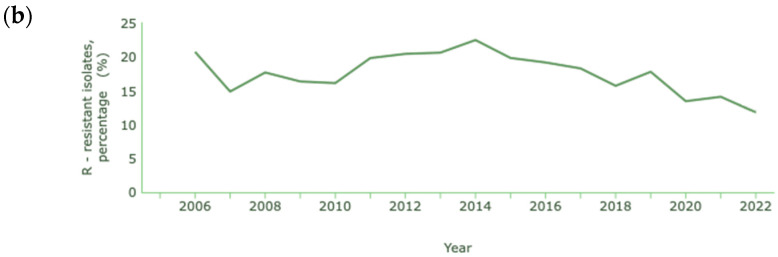
Rate of resistance to carbapenems (**a**) *A. baumannii*; (**b**) *P. aeruginosa*. Dataset provided by ECDC based on data provided by WHO and Ministries of Health from the affected countries (https://atlas.ecdc.europa.eu/public/index.aspx, accessed on 7 March 2024).

In the cases of *A. baumannii* and *P. aeruginosa*, where carbapenem resistance is also common and at a higher rate than *K. pneumoniae*, the percentages reported are lower than in previous years, as shown in Figure 4a and Figure 4b, respectively [131].

The decrease in the resistance of *Acinetobacter* spp. to carbapenems is quite significant, with a reduction from 79.2% to 10.4% between 2012 and 2021. In 2022, unlike previous years, there was a significant increase in cases of resistance, corresponding to 31.1% of cases of infection with *Acinetobacter* spp. In the case of *P. aeruginosa,* a decrease from 20.7% to 11.8% occurred between 2006 and 2022.

**Figure 5:**
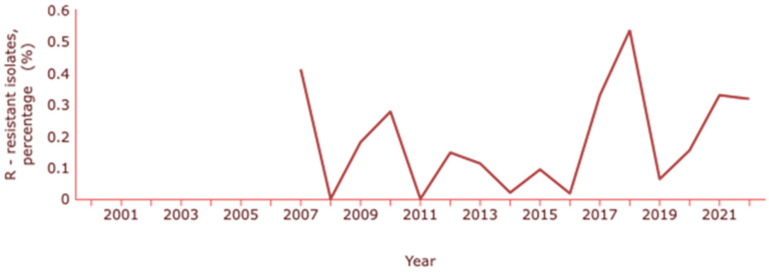
Rate of resistance to carbapenems by *E. coli*. Dataset provided by ECDC based on data provided by WHO and Ministries of Health from the affected countries (https://atlas.ecdc.europa.eu/public/index.aspx, accessed on 7 March 2024).

Concerning *E. coli*, the ECDC indicates a resistance to carbapenems (meropenem and imipenem) of about 0.3% and INSA a resistance percentage of 0.5% [130,132]. The rates of *E. coli* resistance to carbapenems have been changing over the years, with the latest increase coinciding with the pandemic years (Figure 5).

In a national program developed in 2013, the Program for Prevention and Control of Infection and Antimicrobial Resistance (PPCIRA), epidemiological data regarding annual rates of consumption of antibiotics and antimicrobial resistance are reported [133]. In this study, with data reported up to 2020, *Acinetobacter* spp. and *P. aeruginosa* show a reduction in resistance relative to the rest of Europe, being 15% versus 38% and 11% and 12%, respectively [133].

### 5.2. Human Antibiotic Consumption in Portugal

The abusive and incorrect consumption of antibiotics is one of the main contributors to the increase in resistance. For this reason, its monitoring is extremely important, allowing for more focused action in its control [10].

The annual epidemiological report on antimicrobial consumption in the EU/EEA (ESAC-Net) for 2022 published consumption rates of antibiotics dispensed in outpatient clinics and consumed by the community and in the hospital sector in many countries, including Portugal. The data, expressed as defined daily doses (DDD) per 1000 population per day, showed in 2022 a mean total of 19.4 DDD per 1000 inhabitants per day in the EU/EEA. These results show a decrease of 2.8% between 2012 and 2021 but a significant increase when compared to 2020, the year of the beginning of the COVID-19 pandemic in Portugal [14,134,135].

In Portugal, the total consumption of antibiotics was 18.8 DDD per 1000 inhabitants per day, which led to a 2.6% decrease in consumption of antibiotics between 2019 and 2022 [14,135]. Overall, Portugal has a lower consumption of antibiotics when compared to the European average.

The consumption by the community in Portugal decreased by 3.4% until 2021, with a 0.7% increase in 2022, being reported at 17.1 DDD per 1000 inhabitants per day [14,135]. Despite these statistics, when the ratio between the consumption of broad-spectrum and narrow-spectrum antibiotics in the community is analyzed, it increased by 0.6% compared to 2013, with 5.6 DDD per 1000 inhabitants per day. These findings should be taken into consideration by the European authorities and the prescription plans revised so that their use can be reduced, since the use of broad-spectrum antibiotics is a factor in the development of resistance [136].

The total use of antibiotics in the hospital sector in Portugal for the year 2021, similar to the European data, was 1.72 DDD per 1000 inhabitants per day. Compared to 2013, Portugal recorded an increase of 1.4%, contrasting with the decrease of 0.4% in the EU/EEA. Despite these results, the use of carbapenems decreased in Portugal by 2.2% between 2013 and 2022, whereas the EU/EEA reported an increase of 1.8% [135].

Since 2013, the use of carbapenems has been decreasing in the EU and in Portugal, where PPCIRA was introduced, as one of the main objectives is to reduce the prescription and use of carbapenems by 20% compared to the previous year [137].

In 2020, this trend changed with the COVID-19 pandemic. The increase in the use of carbapenems led to the development of new guidelines and targets, and Portugal did not experience a significant increase. This was not the case for some countries, such as Bulgaria and Croatia, which experienced such increases, with rates of 19.6% and 11.7%, respectively [135].

### 5.3. Veterinary Antibiotic Consumption in Portugal

The European Surveillance of Veterinary Antimicrobial Consumption (ESVAC) reports annually data regarding the sales of antimicrobials for use in food-producing animals in 31 countries in Europe and analyzes the trends it has followed since 2010. To better analyze the data, the indicator used is milligrams of active substance sold per population correction unit (mg/PCU), where PCU is applied as a proxy for the size of the food-producing animal population [138]. Collectively, among 25 of the 31 countries that have been reporting data on sales of veterinary medicines since 2011, there has been a decrease of 46.5% mg/PCU by 2021, according to the latest data available [138]. In 2021, Portugal reported total sales of antibiotics for food-producing animals of 149.9 mg/PCU, a decrease of 14.4% compared to 2010 (175.1 mg/PCU). Unfortunately, due to underreporting, the sales numbers for some years, 2010–2014, 2017, and 2019, have been underestimated. In the same year of 2021, the global data reported for all 31 countries was 84.4 mg/PCU. Portugal’s sales rate is 77.6% higher than the average, requiring a review of the measures taken [138]. In general, there is no data on carbapenems, most likely due to the ban on their use in veterinary medicine and food-producing animals.

### 5.4. The Environment and Detection of Antibiotics in Portugal

In Portugal, there is a lack of data on the presence of antibiotics in the environment and their impact. It is assumed that the increase in the use of antibiotics has led to an increase in their deposition in water and soil [139]. The few studies that have been carried out have reported the presence of several antibiotics in Portuguese waters, with penicillins (e.g., amoxicillin) being among the most common [140,141]. Although β-lactams are easily degraded in the environment, their consumption rate is high worldwide, leading to their identification in the environment. In addition to penicillins, there are reports of β-lactamase inhibitors (clavulanic acid and tazobactam) and cilastatin, which is used in combination with imipenem, which could be precursors to carbapenem resistance in the environment [141]. Between 2017 and 2018, the survival of carbapenem-resistant *P. aeruginosa* in Portuguese soils was reported, raising concerns about the spread of antibiotic resistance in soils [142].

## 6. Discussion

Antibiotic resistance is an emerging problem, mostly driven by clinical practice, where bacterial infections caused by resistant pathogens become increasingly difficult to treat. Increasing treatment costs and mortality rates associated with these infections are the main factors contributing to this public health problem.

According to the ECDC, every year, 35,000 people die from antibiotic-resistant infections in the European Union, Iceland, and Norway. Moreover, more than 70% of resistant infections are directly linked to healthcare-associated infections. The number of resistances to last-line treatment antibiotics, such as carbapenems, has been on the rise throughout the years, especially *K. pneumoniae* and *Acinetobacter* spp. [15].

Antibiotic use and abuse play a major role in the development of resistance, as do other factors such as pathogen–host/pathogen–drug interactions, natural selection through survival and emergence of the resistant strains, horizontal gene transfer, mutations, sanitation conditions, and some public health factors [136]. Its consumption is often unduly high in various areas, such as agriculture and aquaculture. In addition to their use in veterinary medicine, they are also resorted to for growth promotion, in a more accelerated manner, as well as disease prevention [10].

In the community, several factors contribute to the spread of resistance, either through food products or through international trade, where possibly endemic bacteria are spread to different countries, as in their preparation [8]. Wastewater is a reservoir of environmental bacteria, and even if treated, it contains genes resistant to antibiotics, causing them to interact and become reservoirs for the evolution and spread of resistant pathogens [143]. These waters are often in agriculture, transmitting the bacteria to fruits and vegetables, which can lead to limiting infections, but with an increased risk in children and immunosuppressed patients, leading to complications [144].

Thus, there is a need for the adoption of the One Health perspective by as many countries as possible, allowing a more complete data collection. With this, improvements in human and animal health, greater and more effective control of bacterial infections, and the transmission of resistance may be promoted by their associations. Some of these measures have shown positive results [145].

The use of carbapenems in the hospital sector had also decreased in Portugal, which could indicate the good management of the measures implemented and also may have had the influence of the COVID-19 pandemic, which had its onset in 2020, both in outpatient and hospital settings [124]. However, attention must be paid to the use of broad-spectrum antibiotics, as they are potentiators of resistance, and their use has been increasing in Portugal [135,136].

Concerning antibacterial resistance, a considerable reduction in the resistance of *Acinetobacter* spp. to carbapenems was evidenced by 2021, while in 2022 there was a rise from 10.4% to 31.1%. This increase may be related to underreporting in previous years due to inter-agency communication difficulties during the COVID-19 pandemic, outbreaks of this pathogen, and/or co-infection in COVID-19 patients [146,147].

This last possibility may also be related to the results of the data on the resistance of *K. pneumoniae*. Although this may have helped, the resistance of *K. pneumoniae* has been increasing sharply since 2014, when outbreaks of this bacterium began to be identified in Portugal [124]. In this case, a revision of the Epidemiological Surveillance Standard on Antimicrobial Resistance is necessary. On 1 June 2023, the Council of the European Union published additional recommendations to intensify the measures already implemented by the EU countries on antibiotic resistance from the One Health perspective [148]. The carbapenem-resistant *K. pneumoniae* needs special attention to take measures in an attempt to prevent an increase in the rate of resistance to carbapenems. These measures can include better control and cleaning of hospital equipment, methods of identifying carbapenemase-producing bacterial strains, and treatment alternatives [149].

Some of these strategies have already been applied, but they do not always prove to be the most effective, which highlights the need for constant monitoring of their effectiveness as well as all the variables that may affect them. Thus, new corrective and, above all, preventive measures should be included to achieve a reduction in resistance rates.

In clinical practice, new approaches to fighting antibiotic-resistant bacteria have been investigated, using combination therapies focusing on different features that normally cause resistance, such as membrane permeability or efflux pumps [150]. New β-lactamase inhibitors have been developed, and their combination with the existing β-lactams has been tested with promising results [151,152]. Vaborbactam is a cyclic boronic acid pharmacophore that enhances the activity of meropenem in resistant strains by inhibiting the production of β-lactamases [153]. Recent data show improved activity against class A carbapenemases, predominantly KPC in members of the *Enterobacteriaceae* (e.g., *K. pneumoniae*, *E. coli*), but limited activity against *Acinetobacter* spp. and *P. aeruginosa* [154]. Imipenem-relebactam and ceftazidime-avibactam combinations have been shown to be effective against resistant bacteria, including multi-drug resistant (MDR) *P. aeruginosa* [155,156]. In addition to its activity against KPC, ceftazidime-avibactam has demonstrated promising results on *Enterobacteriales* carrying *bla*_OXA-48_ [155]. Most of these new drugs and combinations only target serine carbapenemases, but a triple combination of ceftazidime, avibactam, and aztreonam has been able to work effectively against MBLs [157]. A novel cephalosporin, cefiderocol, is being used as an alternative treatment for carbapenem-resistant *A. baumannii*. This antibiotic has demonstrated positive results against a wide range of serine and metallo-β-lactamases [158].

The efficacy of combining polymyxins with different drugs, such as mitotane (an antineoplastic drug), to increase the membrane permeability of Gram-negative bacteria, such as the carbapenem-resistant bacteria *P. aeruginosa*, *A. baumannii,* and *K. pneumoniae*, has been demonstrated and is currently in phase 1 clinical studies [159]. To reduce the activity of efflux pumps, some efflux pump inhibitors (EPIs) have been reported and tested [160].

Other alternative therapies, such as bacteriophage engineering, are also being studied [161]. This therapy is still awaiting approval for clinical use due to some limitations that need further research, such as its immunogenicity, which remains unknown, and the rapid resistance of pathogens after phage treatment [162].

In the animal sector, the regulations need to be reviewed and updated. The last report on the control of antibiotic use in veterinary medicine presented by the Directorate-General for Food and Veterinary Affairs (DGAV) dates from 2018 [163]. In addition, the last publicly published report presents data referring to 2016. The EMA’s reports on the sale of medicines for use in veterinary medicine have highlighted the lack of data for several years [138]. Improved cooperation and more frequent information sharing could facilitate a better understanding of the national landscape in this sector. Consequently, the implementation of measures would be more aligned with the actual needs, potentially leading to enhanced outcomes.

The challenge of antimicrobial resistance across various bacterial families is projected to remain a critical issue for the foreseeable future. Despite the continuous efforts of the scientific community and healthcare institutions, the primary focus should be on political and governmental policies that promote awareness among the general public and healthcare providers, incorporating a One Health approach. It is imperative that all stakeholders collaborate to mitigate the imminent threat of future epidemics, the proliferation of “superbugs,” and the potential ineffectiveness of current protocols and measures.
